# Multimorbidity and quality of life: a closer look

**DOI:** 10.1186/1477-7525-5-52

**Published:** 2007-08-06

**Authors:** Martin Fortin, Marie-France Dubois, Catherine Hudon, Hassan Soubhi, José Almirall

**Affiliations:** 1 Department of Family Medicine, Sherbrooke University, Sherbrooke, Canada; 2 Unité de médecine de famille, Centre de Santé et de services sociaux de Chicoutimi, Chicoutimi, Canada; 3 Department of Community Health Sciences, Sherbrooke University, Sherbrooke, Canada; 4 Research Center on Aging, Sherbrooke Geriatric University Institute, Canada

## Abstract

**Background:**

The presence of multiple chronic conditions is associated with lower health related quality of life (HRQOL). Disease severity also influences HRQOL. To analyse the effects of all possible combinations of single diseases along with their severity on HRQOL seems cumbersome. Grouping diseases and their severity in specific organ domains may facilitate the study of the complex relationship between multiple chronic conditions and HRQOL. The goal of this study was to analyse impaired organ domains that affect the most HRQOL of patients with multiple chronic conditions in primary care and their possible interactions.

**Methods:**

We analysed data from 238 patients recruited from the clientele of 21 family physicians. We classified all chronic conditions along with the measure of their severity into the 14 organ domains of the Cumulative Illness Rating Scale (CIRS). Patients also completed the 36-item Medical Outcomes Study questionnaire (SF-36). One-way analyses of variance were performed to study the relationship between the severity score for each CIRS domain and both physical component summary (PCS) and mental component summary (MCS) of HRQOL. Two-way analyses of variance were conducted to investigate the significance of possible organ domains interactions. Variables involved in significant bivariate relationships or interactions were candidates for inclusion in a multivariate model. Five additional variables were included in the multivariate model because of their possible confounding effect: perceived social support, age, education, perceived economic status and residual CIRS.

**Results:**

Significant differences in the PCS (p < 0.01) were found in 12 of the 14 CIRS organ domains. A significant difference in MCS was found only in the Psychiatric domain. In the multivariate analysis for the PCS, the CIRS domains Musculoskeletal, Neurological, and Psychiatric, had an independent direct impact on PCS while the Upper gastrointestinal, Vascular, Cardiac and Respiratory domains were involved in interactions. A multivariate model was not necessary for the mental component.

**Conclusion:**

Vascular, Upper gastrointestinal and Musculoskeletal systems have strong negative effects on HRQOL. Among combinations of systems, the respiratory and cardiac combination is of particular concern because of a synergistic negative effect. This study paves the way for a future study with a bigger sample that could yield a model of wider generalizability.

## Background

Goals of caring for patients with a chronic condition are to enhance their functional status, minimize symptoms, control pain, reduce disability, and prolong life through secondary prevention [1]. Patients with multiple chronic conditions or multimorbidity are the rule rather than the exception in primary care [2]. For family physicians to pursue the same care goals for patients with multimorbidity, they have to develop a partnership with patients and set priorities to address patients' health care needs with the aim of increasing their life span while maintaining an optimal quality of life [3,4].

Health-related quality of life (HRQOL) provides a multidimensional perspective that encompasses a patient's physical, emotional, and social functioning [5]. In general, patients with more than one comorbid condition report the poorest level of HRQOL [6], but some chronic conditions are more strongly associated with poor HRQOL than others. The severity of the individual conditions is also a factor influencing HRQOL[6]. Knowledge of the conditions that potentially have a greater impact on the HRQOL may help family physicians focus their attention on those conditions. The combined influence of pairs of chronic medical conditions on HRQOL have received some attention, [7-10] but patients with multimorbidity often present more than two chronic medical conditions and have often multiple body systems involved [2].

Disease interactions known to affect HRQOL include, among others, diabetes with hypertension, heart disease, arthritis, obesity and respiratory problems; arthritis with heart disease, hypertension, pulmonary diseases and obesity [7-10]. To make an analysis using individually all possible chronic conditions, their possible combinations and effects on the HRQOL seems cumbersome. Grouping diseases and their severity in specific organ domains may facilitate the understanding of the complex relationship between multiple chronic conditions and HRQOL. We used the Cumulative Illness Rating Scale (CIRS) in a previous study to document the association between HRQOL and multimorbidity (presence of two or more chronic conditions). We showed that the CIRS was a better predictor of HRQOL than a simple count of chronic diseases [6]. The CIRS is a comprehensive comorbidity index which takes into account the severity of the conditions using a scoring mechanism that encompasses 14 anatomical domains (Cardiac, Vascular, Hematological, Respiratory, Ophtalmological-ORL, Upper gastrointestinal, Lower gastrointestinal, Hepatic-pancreatic, Renal, Genitourinary, Musculoskeletal-tegumental, Neurological, Endocrine-metabolic-breast, and Psychiatric) [11-14]. The total CIRS score is the sum of severity scores assigned to each organ domain. So far, we have only used the total CIRS score in our studies [6,15,16]. We hypothesize that the individual organ domain score may further our understanding of the complex relationship between multimorbidity and HRQOL.

The goal of this study was therefore to analyse which impaired organ domains affect the most HRQOL of patients with multiple chronic conditions in primary care and their possible interactions.

## Methods

We used data collected in a group of 238 adult primary care patients (age 18 or older) recruited from the clientele of 21 family physicians in the Saguenay region, Canada, who participated in a study on HRQOL [6]. Details of the selection process are described elsewhere [6]. In brief, we randomly selected patients from 980 patients who had also been selected at random for a prevalence study on multimorbidity [2]. Our goal was to recruit patients to complete five CIRS groups (quintiles) of 60 patients each with different levels of multimorbidity. Of the 419 patients we tried to contact by phone, 66 could not be reached, despite repeated attempts. Of the remaining 353 patients, 238 agreed to participate (participation rate of 67%). Patients who refused to participate did not have time (66 patients), or were not interested (42 patients); 7 patients were judged to be in an acute state of illness and were not included.

From an exhaustive chart review, we extracted a comprehensive list of diagnoses of all chronic conditions for every patient after informed consent. For this review, the information contained in the medical chart was considered valid and complete. We then scored the Cumulative Illness Rating Scale (CIRS) [17].

Each domain of the CIRS is scored from 0 to 4 as follows: 0, no problem; 1, minor current problem or significant history; 2, morbidity or moderate discomfort, requiring treatment; 3, severe problem, constant significant discomfort, chronic problem difficult to control; 4, extremely severe problem, requiring immediate treatment, organ failure or severe functional impairment. The theoretical score ranges from 0 to 56, but scores close to the highest value are incompatible with life. The CIRS has been shown to be valid and reliable for its use in primary care [18].

Patients completed the French version of the self-administered 36-item short form of the Medical Outcomes Study questionnaire (SF-36) that is well known and validated to assess HRQOL [19]. The SF-36 comprises 8 multi-item scales divided into 2 main groups: physical and mental aspects of quality of life. Two summary scores for each group are obtained through a weighted sum of these scales. For all scales and both summary scales, lower scores indicate lower HRQOL. In this study, we used the physical component summary (PCS) and the mental component summary (MCS) in the analyses.

Data for potential confounders (age, education, self-perceived economic status and self-perceived social support) were also collected. Self-perceived social support was measured with the Social Provisions Scale [20].

The research ethics board of the Centre de santé et de services sociaux de Chicoutimi approved this study.

### Statistical analysis

Characteristics of the participants were first summarized using means and standard deviations or percentages. We then examined the proportion of subjects falling into each severity level for the 14 domains covered by the CIRS. Severity levels with low prevalence (less than 10 subjects) were combined with the adjacent level for subsequent analyses.

For each domain of the CIRS, one-way analyses of variance (ANOVA) were first performed to study the relationship between severity and both physical (PCS) and mental (MCS) components of quality of life, ignoring the effect of other domains. Severity levels with non significant differences in quality of life were combined. Two-way analyses of variance were conducted to investigate the significance of possible interactions based on disease interactions previously identified in the literature [7-10] or in our practice. The CIRS domains included in the analysis of interactions for the PCS were: Cardiac, Vascular, Respiratory, Upper gastrointestinal, Musculoskeletal, Neurological, Endocrine. Variables involved in bivariate relationships or interactions significant at the 0.15 level were candidates for inclusion in a multivariate model.

Five additional variables were included in the multivariate model because of their possible confounding effect: perceived social support, age, education, perceived economic status and residual CIRS [6]. The latter was defined as the sum of severity scores for the systems excluded from the model. A backward elimination procedure was then applied to eliminate variables which cease to be statistically significant (p > 0.05) in the presence of others. However, variables with a significant interaction term were kept in the model, regardless of their significance level to ensure the hierarchy principle. Analyses were performed with SAS (SAS Institute Inc., Cary, NC), version 8.02.

## Results

**Figure 1:**
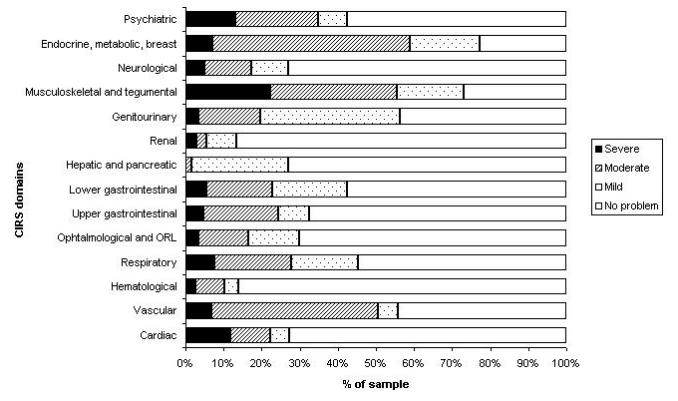
**Percentage of sample in each severity level**. The percentage of sample in each severity level is shown for the 14 domains covered by the CIRS. It should be noted that The CIRS assigns a value from 0 to 4 to determine a severity score for each domain (0 = no problem; 1 = mild; 2 = moderate; 3 = severe; 4 = extremely severe). Due to the small number of subjects, we combined the "extremely severe" CIRS category with the "severe" category.

Characteristics of the 238 participants are reported elsewhere [6]. On average, participants were 59.0 ± 14.3 years old (mean ± SD), had 5.3 ± 2.8 diagnoses and a CIRS score of 10.3 ± 5.7; 29% were male. Summary scores of SF-36 were 42.22 ± 12.28 and 50.37 ± 10.21 for the physical (PCS) and mental (MCS) components respectively. Figure 1 shows the proportion of each severity level for the 14 domains covered by the CIRS. Due to the small number of subjects, we combined the "extremely severe" CIRS category with the "severe" CIRS category.

Results from the bivariate analyses of the 14 domains of the CIRS with the PCS are shown in Table 1. Significant differences in the PCS among CIRS severity categories (p < 0.01) were found in 12 CIRS domains. Among these domains, only 2 subgroups of disease severity could be differentiated according to the PCS in 9 CIRS domains. Three subgroups of disease severity could be differentiated in the 3 remaining CIRS domains (Respiratory, Musculoskeletal and Endocrine).

**Table 1:** Relationship between the physical component summary (PCS) of SF-36 and severity of problems in each domain of the CIRS

**Degrees of severity by domain of CIRS**	**PCS (mean ± sd)**	**p value**	η^2^
**Cardiac**			
No problem/Mild/Moderate	43.2 ± 11.8	0.0004	5.19%
Severe	39.2 ± 12.9		
**Vascular**			
No problem	46.7 ± 11.4	<0.0001	10.83%
Mild/Moderate/Severe	38.6 ± 11.8		
**Haematological**			
No problem	42.7 ± 12.1	0.1221	1.01%
Mild/Moderate/Severe	39.2 ± 12.9		
**Respiratory**			
No problem/Mild	44.7 ± 11.7	<0.0001	12.14%
Moderate	37.5 ± 10.9		
Severe	31.2 ± 12.4		
**Ophthalmologic and ORL**			
No problem/Mild	43.4 ± 12.1	0.0013	4.32%
Moderate/Severe	36.5 ± 11.6		
**Upper gastrointestinal**			
No problem/Mild	44.3 ± 11.6	<0.0001	9.17%
Moderate/Severe	35.7 ± 12.2		
**Lower gastrointestinal**			
No problem/Mild	43.4 ± 12.1	0.0050	3.30%
Moderate/Severe	38.1 ± 12.2		
**Hepatic and pancreatic**			
No problem	42.9 ± 11.8	0.1483	0.89%
Mild/Moderate	40.3 ± 13.3		
**Renal**			
No problem/Mild	42.6 ± 12.2	0.0457	1.69%
Moderate/Severe	35.6 ± 12.3		
**Genitourinary**			
No problem	44.5 ± 12.2	0.0101	2.78%
Mild/Moderate/Severe	40.4 ± 12.1		
**Musculoskeletal**			
No problem	48.8 ± 9.2	<0.0001	16.44%
Mild/Moderate	42.0 ± 12.2		
Severe	34.6 ± 11.2		
**Neurological**			
No problem	44.5 ± 11.6	<0.0001	9.72%
Mild/Moderate/Severe	35.9 ± 11.9		
**Endocrine, metabolic, breast**			
No problem/Mild	44.8 ± 11.6	0.0014	5.44%
Moderate	41.3 ± 11.9		
Severe	33.9 ± 14.8		
**Psychiatric**			
No problem/Mild	44.5 ± 11.6	<0.0001	6.58%
Moderate/Severe	37.9 ± 12.4		

In the analyses of interactions, only 2 were significant: the Cardiac-Respiratory and the Vascular-Upper gastrointestinal. Details are shown in Table 2.

**Table 2:** Significant results of the analysis of interactions among CIRS domains

**Interaction**	**PCS (mean ± sd)**	**p value**	η^2^
**Cardiac – Respiratory**			
Low cardiac^† ^and low respiratory^‡^	44.8 ± 11.5	<0.0001	14.99%
Severe cardiac and low respiratory^‡^	43.4 ± 13.4		
Low cardiac^† ^and high respiratory^§^	38.4 ± 11.2		
Severe cardiac and high respiratory^§^	27.0 ± 8.0		
**Vascular – Upper gastrointestinal**			
No vascular and low upper gastro^‡^	49.2 ± 9.3	<0.0001	20.12%
Vascular^¥ ^and low upper gastro^‡^	39.9 ± 11.7		
No vascular and high upper gastro^§^	36.1 ± 13.5		
Vascular^¥ ^and high upper gastro^§^	35.5 ± 11.6		

^† ^No problem, mild or moderate problem

^‡ ^No problem or mild problem

^§ ^Moderate or severe

^¥ ^Mild, moderate or severe

In the bivariate analyses of the 14 domains of the CIRS with the MCS, a significant difference in MCS was found only between 2 severity subgroups in the Psychiatric domain: no problem/mild/moderate vs. severe (p < 0.001, η^2 ^= 6.54%). Therefore, a multivariate model was not necessary for the mental component.

Results from the multivariate analysis for the PCS are shown in Table 3. The CIRS domains Musculoskeletal, Neurological, and Psychiatric, have an independent direct impact on PCS while the Upper gastrointestinal, Vascular, Cardiac and Respiratory domains are involved in interactions. The chronic conditions most commonly found in these domains were, respectively: stomach or duodenal ulcers and gastro-oesophageal reflux; hypertension; coronary artery disease and chronic heart failure; chronic obstructive pulmonary disease and asthma. The coefficients in Table 3 allow calculation of an estimate of the PCS for subjects with different problems. For example, for a subject with no problems in the musculoskeletal, neurological and vascular domains and no or mild problems in the psychiatric and upper gastrointestinal domains, the PCS for moderate or severe respiratory problem and severe cardiac problem can be estimated as:

**Table 3:** Reduced multivariate model for the physical component summary (PCS) of the SF-36

**Variable**	**Parameter Estimate**	**Standard error**	**p value**
Intercept	54.62	1.42	<0.0001
Mild or moderate Musculoskeletal problem (**Mus1,2**)	-3.77	1.53	0.0145
Severe Musculoskeletal problem (**Mus3,4**)	-8.11	1.92	<0.0001
Mild, moderate or severe Neurological problem (**Neu1,2,3,4**)	-3.84	1.50	0.0111
Moderate or severe Psychiatric problem (**Psy2,3,4**)	-4.06	1.40	0.0041
Severe Cardiac problem (**Car3,4**)	-1.85	2.83	0.5142^†^
Moderate or severe Respiratory problem (**Res2,3,4**)	-3.11	1.62	0.0564^†^
**Car3,4 **and **Res2,3,4**	-8.85	4.07	0.0307
Moderate or severe Upper gastrointestinal problem (**Upg2,3,4**)	-8.98	2.47	0.0003
Mild, moderate or severe Vascular problem (**Vas1,2,3,4**)	-6.58	1.49	<0.0001
**Upg2,3,4 **and **Vas1,2,3,4**	+8.08	3.10	0.0097

^† ^kept in the model because involved in an interaction

PCS = 54.62 + (-3.11) + (-1.85) + (-8.85) = 40.8

For subjects with no problems in the musculoskeletal and neurological domains, no or mild problems in the psychiatric and respiratory domains and no severe cardiac problems, the PCS for moderate or severe upper gastrointestinal problem and mild, moderate or severe vascular problem can be estimated as:

PCS = 54.62 + (-8.98) + (-6.58) + (+8.08) = 47.1

**Figure 2:**
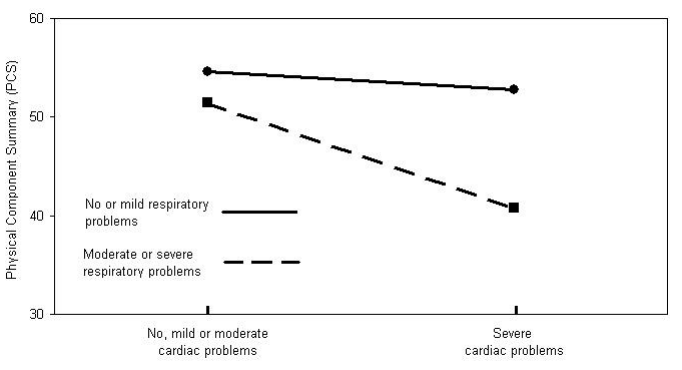
**Illustration of the interaction between Cardiac and Respiratory CIRS domains**. The figure illustrates the interaction between Cardiac and Respiratory domains for subjects with no problems in the musculoskeletal, neurological and vascular domains and no or mild problems in the psychiatric and upper gastrointestinal domains.

**Figure 3:**
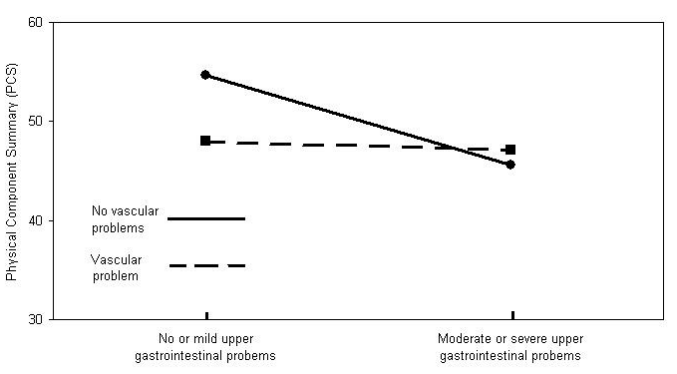
**Illustration of the interaction between Upper gastrointestinal and Vascular CIRS domains**. The figure illustrates the interaction between Upper gastrointestinal and Vascular CIRS domains for subjects with no problems in the musculoskeletal and neurological domains, no or mild problems in the psychiatric and respiratory domains and no severe cardiac problems.

Graphical displays of these interactions are shown in Figures 2 and 3.

## Discussion

In the present study, we have been able to develop a multivariate model relating the physical aspect of HRQOL (as measured by the PCS of the SF-36) and all chronic conditions – grouped by body systems – of patients with multimorbidity, while taking into account the degree of severity of the health impairments evaluated with the CIRS. Although small in size, this is the first study evaluating the impact of systems involvement and their combination on HRQOL.

The summary scales PCS and MCS were chosen to represent HRQOL in this study because they have been shown to be among the most valid SF-36 scales for measuring physical health and mental health, respectively [21]. Also, confidence intervals around individual scores are much smaller for the two summary measures than for the eight scales (+/- 6–7 points versus +/- 13–32 points, respectively) [21].

In our multivariate model, all variables have a different weight on the resultant PCS. Impairments in all the CIRS domains that remained in the multivariate model have a negative coefficient (Table 3). This means that, according to the model, these impairments have a negative impact on PCS. The impairment with the highest direct impact was severe Musculoskeletal problem reducing PCS by 8 points (Table 3). This latter result is consistent with reports associating musculoskeletal disorders with large losses of HRQOL [22,23]. This is also congruent with the clinical experience of family doctors. Diseases included in the musculoskeletal system are any form of arthritis, chronic low back pain and any rheumatologic or orthopaedic chronic problems.

Apart from being additive (the sum of the independent effect of each condition approximates their combined effect), the combined influence of pairs of chronic conditions on the HRQOL can also be synergistic (the combined effect is greater than the sum of the independent effect of each condition) or subtractive (the combined effect is smaller than the sum of the independent effect of each condition) [7-10,24].

We found a synergistic effect between respiratory and cardiac systems which replicates results previously reported by Rijken *et al.*[10]. They found that patients with cardiovascular disease reported equal levels of PCS as patients with other chronic diseases considered in their study. However, patients with respiratory disease reported worse PCS than patients with other chronic disease, and the combination of cardiovascular and respiratory diseases had a negative synergistic effect on the PCS [10]. Our data confirm the same synergistic effect of these systems on PCS since, in our multivariate model, cardiac problems are not significant, respiratory problems only approach significance and their interaction is highly significant i.e. their combined effect decreases more the PCS than the sum of the independent negative effect of each condition. This is congruent with what we see in our clinical practice. A possible explanation is the common shortness of breath that is present when both systems are involved.

We found a subtractive interaction between moderate or severe Upper gastrointestinal problem and mild, moderate or severe Vascular problem. The independent effect of each condition is quite important in reducing physical functioning, but their combined effect is smaller than the sum of the independent effects (Figure 3). We cannot provide a logical explanation for this finding. This will have to be confirmed and explored in more details.

The literature reports other additive and synergistic interactions of pairs of diseases such as diabetes-hypertension (Endocrine-Vascular domains in the CIRS), diabetes-heart disease (Endocrine-Heart), diabetes-arthritis (Endocrine-Musculoskeletal) and arthritis-respiratory disease (Musculoskeletal-Respiratory) that were not found to be significant in our analysis of interactions by CIRS domains [7-10,24]. Chronic conditions in CIRS domains or interactions not included in the reduced multivariate model should not be interpreted as domains in which health problems do not have an impact on HRQOL or that those interactions are not important for individual patients. It only means that the affected domains and their interactions included in the model are those with a greater impact on the physical functioning in the sample of subjects of this study.

It is worth noting that the Psychiatric domain of the CIRS remained significant in the multivariate model for the physical functioning. Whereas this domain is frequently excluded from studies relating comorbidity or multimorbidity with HRQOL [25], our results highlight its importance in such studies and are in agreement with those reported by Rijken *et al.*[10].

Rijken *et al.* also assessed the joint effects of a limited number of somatic chronic diseases and found that, in general, the MCS was not related to any of the diseases evaluated with the exception of a negative effect of thyroid dysfunction [10]. In our study, the bivariate analysis showed a significant relationship of the MCS only with the Psychiatric domain. As we evaluated the levels of severity, we found that the significant difference was at the "Severe" level.

Knowledge of the estimates of the relative impact of individual chronic diseases on HRQOL is important for decision-makers to better plan and allocate resources for research, training and health care [4]. For the primary care physician, for whom multimorbidity implies a list of health problems, the knowledge of specific systems or their combinations that have a greater impact on the patient's HRQOL is essential for individual patient care and can be helpful to establish priorities in management decisions [24]. Given that patients with multimorbidity represent the majority in primary care, this knowledge is also important for public health purposes.

This study was based on the analysis of an existing database, and its main limitation is the relatively small sample of subjects. This limitation is directly related to the effect of the intentional sampling process to recruit patients that represented five CIRS groups, each with different levels of multimorbidity. This sampling process by itself was not expected to affect the relationships in our analyses but produced its effect through the size of the sample we obtained. The number of subjects per severity level varied considerably. This forced us to group subjects with problems of different levels of severity. Also, a number of diseases within the CIRS domains may not be represented. A bigger sample would have given more power to the statistical analyses and any application of the multivariate model at its present state should be made with caution. Another limitation of the study is that the list of diagnosis was based on chart review of all diagnoses already made by the primary care physician and no attempt was made to assess whether the diagnoses were correct. This method has the limitation that conditions frequently misdiagnosed such as asthma and chronic obstructive pulmonary disease, or frequently undiagnosed such as mental disorders were not verified. Due to all the limitations listed above, we consider that any generalizability of the model at its present state should be made with caution. It might be applicable only to the primary care clientele of our region.

Unlike previous studies, we did not analyze the combined effects from a pre-determined list of diseases; instead, we included in the corresponding CIRS domains all the chronic conditions found in the subjects. This approach is pragmatic and directly in line with clinical practice. Nevertheless, the combined influence of more than two chronic conditions pertaining to more than two systems on the HRQOL can be expected to be more complex than that of a pair of diseases and consequently, caution is mandatory in the interpretation of the study results.

## Conclusion

With this study we have demonstrated the feasibility of developing a multivariate model for the physical functioning aspect of the HRQOL applicable to patients with multimorbidity that takes into account all the chronic diseases (grouped by organ domains) and their level of severity, while controlling for age, education, perceived social support and economic status. Simple disease count as a measure of multimorbidity has a weak correlation with HRQOL; grouping diseases by organ domains which take into account disease severity is a practical alternative to consider individually each condition and its severity. Vascular, Upper gastrointestinal and Musculoskeletal systems have strong negative effects on HRQOL. Among combinations of systems, the respiratory and cardiac combination is of particular concern because of a synergistic negative effect. This study paves the way for a future study with a bigger sample that could yield a model of wider generalizability.

## Competing interests

The author(s) declare that they have no competing interests.

## Authors' contributions

MF participated in the conception and design of the study, supervised data collection and analysis and drafted the manuscript. M-FD participated in the conception and design of the study, performed the statistical analysis and helped draft the manuscript. CH participated in the design of the study critically reviewed the manuscript. HS participated in data analysis and critically reviewed the manuscript. JA participated in the data analysis and helped draft the manuscript. All authors gave their final approval of the version of the manuscript submitted for publication.
